# Crystal structure of stable protein CutA1 from psychrotrophic bacterium *Shewanella* sp. SIB1

**DOI:** 10.1107/S0909049510028669

**Published:** 2010-11-12

**Authors:** Aya Sato, Sonoko Yokotani, Takashi Tadokoro, Shun-ichi Tanaka, Clement Angkawidjaja, Yuichi Koga, Kazufumi Takano, Shigenori Kanaya

**Affiliations:** a Department of Material and Life Science, Osaka University, 2-1 Yamadaoka, Suita 565-0871, Japan; b JST-CREST, 2-1 Yamadaoka, Suita 565-0871, Japan

**Keywords:** CutA1, *Shewanella* sp. SIB1, crystal structure, thermal denaturation, trimeric structural motif

## Abstract

CutA1 is widely found in bacteria, plants and animals, including humans. The functions of CutA1, however, have not been well clarified. It is known that CutA1s from *Pyrococcus horikoshii*, *Thermus thermophilus* and *Oryza sativa* unfold at temperatures remarkably higher than the growth temperatures of the host organisms. In this work the crystal structure of CutA1 from the psychrotrophic bacterium *Shewanella* sp. SIB1 (SIB1–CutA1) in a trimeric form was determined at 2.7 Å resolution. This is the first crystal structure of a psychrotrophic CutA1. The overall structure of SIB1–CutA1 is similar to those of CutA1 from *Homo sapiens*, *Escherichia coli*, *Pyrococcus horikoshii*, *Thermus thermophilus*, *Termotoga maritima*, *Oryza sativa* and *Rattus norvergicus*. A peculiarity is observed in the β2 strand. The β2 strand is divided into two short β strands, β2a and β2b, in SIB1–CutA1. A thermal denaturation experiment revealed that SIB1–CutA1 does not unfold completely at 363 K at pH 7.0, although *Shewanella* sp. SIB1 cannot grow at temperatures exceeding 303 K. These results indicate that the trimeric structural motif of CutA1 is the critical factor in its unusually high stability and suggest that CutA1 needs to maintain its high stability in order to function, even in psychrotrophs.

## Abstract 2

The crystal structure of CutA1 from the psychrotrophic bacterium *Shewanella* sp. SIB1 in a trimeric form was determined at 2.7 Å resolution. This is the first crystal structure of a psychrotrophic CutA1.

## 1. Introduction

CutA1 is universally distributed in bacteria, plants and animals. CutA1 from *Homo sapiens* (Hs-CutA1) appears to be necessary for the localization of acetylcholinesterase at the cell surface (Navaratnam *et al.*, 2000 ▶; Perrier *et al.*, 2000 ▶). Recently it has been shown that mammalian CutA1 affects the folding, oligomerization and secretion of acetylcholinesterase (Liang *et al.*, 2009 ▶). In addition, homologues to bacterial CutA1 belong to an operon involved in copper tolerance. CutA1 from *Escherichia coli* (Ec-CutA1) is related to the copper-tolerance mechanism (Fong *et al.*, 1995 ▶). The crystal structures of several CutA1s have been determined: Hs-CutA1, Ec-CutA1, CutA1s from *Thermus thermophilus* (Tt-CutA1), *Oryza sativa* (Os-CutA1), *Pyrococcus horikoshii* (Ph-CutA1), *Rattus norvergicus* (Rn-CutA1) and *Termotoga maritima* (Tm-CutA1) (Arnesano *et al.*, 2003 ▶; Tanaka *et al.*, 2004 ▶; Savchenko *et al.*, 2004 ▶; Bagautdinov *et al.*, 2008 ▶). The trimer structures of CutA1 are quite similar, even between mammalian and bacterial CutA1s, suggesting a functional link between them. The specific functions of CutA1, however, have not been well clarified.

It has been reported that CutA1 from a hyperthermophile, Ph-CutA1, has a denaturation temperature of almost 423 K (Tanaka *et al.*, 2006 ▶), which is the highest temperature reported in globular proteins. Other CutA1s, such as Tt-CutA1 and Os-CutA1, also unfold at remarkably higher temperatures than the growth temperatures of the host organisms (Sawano *et al.*, 2008 ▶). The present state of knowledge cannot explain why CutA1 has such unusually high stability. Moreover, it is not known whether CutA1 from psychrophiles and psychrotrophs, in which most proteins are unstable, is remarkably stable or not.

**Figure 1:**
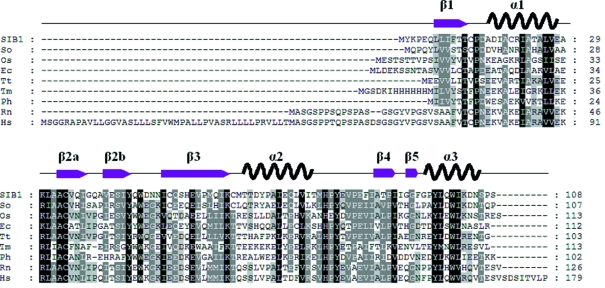
Amino acid sequence alignment of SIB1–CutA1 with other CutA1s. The secondary structural elements of SIB1–CutA1 appear above the sequences. Highly conserved residues are highlighted in black, and conserved residues are in grey. So: *Shewanella oneidensis*; Os: *Oryza sativa*; Ec: *E scherichia coli*; Tt: *Thermus thermophilus*; Tm: *Termotoga maritime*; Ph: *Pyrococcus horikoshii*; Rn: *Rattus norvergicus*; Hs: *Homo sapiens*.

*Shewanella* sp. strain SIB1 is a psychrotrophic bacterium that grows rapidly at 293 K (Kato *et al.*, 2001 ▶). It cannot grow at temperatures exceeding 303 K, but does grow at temperatures as low as 273 K. FKBP22 and ribonuclease HI from *Shewanella* sp. SIB1 exhibit enzymatic properties characteristic of cold-adapted enzymes (Suzuki *et al.*, 2004 ▶; Ohtani *et al.*, 2001 ▶). CutA1 from this strain (SIB1–CutA1) contains 108 amino acid residues and shares 30, 25 and 39% identity with Ec-CutA1, Ph-CutA1 and Hs-CutA1. Fig. 1 ▶ presents the amino acid sequence alignment of CutA1s from several organisms. In this work we cloned the gene encoding CutA1 from *Shewanella* sp. SIB1, overexpressed it in *E. coli*, purified the recombinant protein and crystallized it. We then determined the crystal structure of SIB1–CutA1 and measured its thermal stability. The overall structure of SIB1–CutA1 is similar to that of other CutA1s. A thermal denaturation experiment revealed that SIB1–CutA1 does not completely unfold at 363 K. Based on these results, we discuss the robustness of the CutA1 structure from the viewpoint of its structure and function.

## 2. Materials and methods

### 2.1. Cloning

Genomic DNA of *Shewanella* sp. SIB1 was prepared as previously described (Suzuki *et al.*, 2005 ▶) and was used as a template to amplify part of the SIB1–CutA1 gene by polymerase chain reaction (PCR). Plasmid pETCutA1 for overproduction of SIB1–CutA1 was constructed by ligating the DNA fragment, which was amplified by PCR using a cloned DNA fragment containing the SIB1–CutA1 gene as a template, into the *Nde*I-*Bam*HI sites of pET25b.

### 2.2. Overproduction and purification

*E. coli* BL21(DE3) was transformed with pETCutA1 and grown at 310 K. When *D*_600_ reached 0.6, 1 m*M* IPTG was added to the culture medium and cultivation was continued at 310 K for 4 h. The cells were harvested by centrifugation, disrupted by sonication, and heat-treated at 333 K for 10 min. The supernatant was dialyzed against 50 m*M* Tris-HCl at pH 8.0 and applied to the HiPrep DEAE column. The protein was eluted from the column with a linear gradient of 0 to 1.0 *M* NaCl. The purity was analyzed by SDS-PAGE. The protein concentration was determined from UV absorption using an A280 value of 1.5 for a 0.1% solution (Goodwin & Morton, 1946 ▶).

### 2.3. Crystallization, data collection and structure determination

SIB1–CutA1 was crystallized using the sitting-drop vapour-diffusion method with 2.0 *M* Na/K phosphate and 100 m*M* acetate at pH 4.5. The crystals belonged to space group *P*4_1_2_1_2 and contained six protein molecules per asymmetric unit. X-ray diffraction data were collected at a wavelength of 1.0 Å from the beamline BL38B1 station at SPring-8. Diffraction data were processed using the *HKL2000* program (Otwinowski & Minor, 1997 ▶). The structure was initially solved by molecular replacement with the structure of Ec-CutA1 (Protein Data Bank code 1naq) using the *MOLREP* program (Vagin & Teplyakov, 2000 ▶). Model building and refinement of the structure were completed using the *REFMAC* and *Coot* programs (Murshudov *et al.*, 1997 ▶; Emsley & Cowtan, 2004 ▶). The statistics for data collection and refinement are summarized in Table 1 ▶. The figures were prepared using *PyMol* (DeLano, 2004 ▶). Coordinates and structure factors for the structure of SIB1–CutA1 have been deposited in the Protein Data Bank under accession code 3ahp.

### 2.4. Circular dichroism (CD) spectroscopy and thermal denaturation

The measurements of CD spectra and thermal denaturation were made on a J-725 automatic spectropolarimeter. The mean residue ellipticity, θ, which has units of degrees cm^2^ dmol^−1^, was calculated. The thermal denaturation curves of SIB1–CutA1 were determined by measuring the change in circular dichroism at 220 nm in 0.1 mg ml^−1^ in 10 m*M* Na phosphate at pH 7.0, 1 m*M* EDTA, 1 m*M* DTT and 5% glycerol in a 2 mm cuvette. The heating rate was 60 K h^−1^. The thermal denaturation of SIB1–CutA1 in this condition was reversible up to 368 K. For CD spectra measurements the buffers were 10 m*M* Na phosphate at pH 7.0 or 10 m*M* Na acetate at pH 5.0, 1 m*M* EDTA, 1 m*M* DTT and 5% glycerol.

## 3. Results and discussion

### 3.1. Structure of SIB1–CutA1

**Figure 2:**
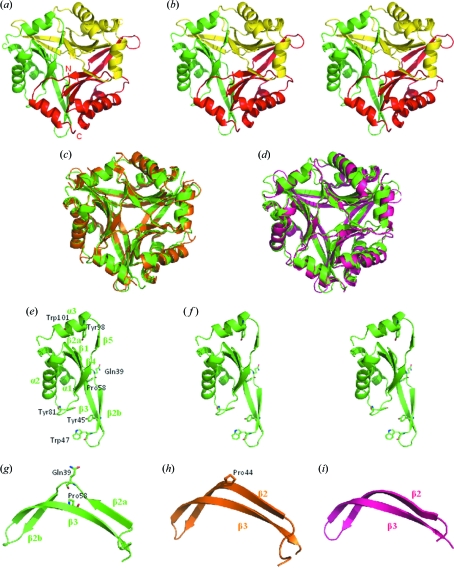
(*a*–*b*) Ribbon diagrams of the trimeric structures of SIB1–CutA1 with secondary structure elements. In the trimeric structure each monomer is coloured differently. (*b*) Stereo drawing. (*c*–*d*) Superimposed structures of SIB1–CutA1 (green) with Ec-CutA1 (orange) and Ph-CutA1 (purple). (*e*–*f*) Ribbon diagrams of the monomeric structure of SIB1–CutA1 with secondary structure elements. (*e*) Residues described in the text are shown. (*f*) Stereo drawing. (*g*–*i*) Close-up views of β2 (β2a and β2b) and β3. Residues described in the text are shown. (*g*) SIB1–CutA1. (*h*) Ec-Cut-A1. (*i*) Ph-CutA1.

The trimeric structure of SIB1–CutA1 was refined at a resolution of 2.7 Å [Figs. 2(*a*) and 2(*b*) ▶]. The overall structure of SIB1–CutA1 resembles that of other homologous CutA1s [Figs. 2(*c*) and 2(*d*) ▶]. The root-mean-square deviations (r.m.s.d.) of the C^α^ atoms for Ec-CutA1 and Ph-CutA1 against SIB1–CutA1 are 1.00 and 1.08 Å as a monomer, and 1.11 and 1.17 Å as a trimer. The r.m.s.d. of the C^α^ atoms between any pair of subunits of SIB1–CutA1 are 0.50–0.58Å. Each subunit (*i.e.* subunit *A*, *B* or *C*) consists of three α helices and six β strands [Figs. 2(*e*) and 2(*f*) ▶], although the monomers of the other CutA1s have three α helices and five β strands. The main difference is observed in the β2 strand. The β2 strand is divided into two short β strands, β2a and β2b, in SIB1–CutA1 (Fig. 2*g* ▶). Hs-CutA1, Ec-CutA1, Tt-CutA1, Os-CutA1 and Rn-CutA1 have a conformational kink in the β2 strand (Fig. 2*h* ▶), whereas Ph-CutA1 and Tm-CutA1 from hyperthermophiles do not exhibit any kink (Fig. 2*i* ▶). This kink results from an insertion of Pro residue into the β2 strand. A Pro deletion variant of Ec-CutA1 increases the stability (K. Yutani, personal communication). In the case of SIB1–CutA1 from a psychrotrophic bacterium, Gln residue (Gln39) is inserted into the β2 strand and the β2 strand is divided into two short β strands. CutA1 from the psychrotrophic bacterium *Shewanella oneidensis* MR-1 (So-CutA1) also has an insertion of Ala residue in the β2 strand (GenBank entry NC_004347) (Fig. 1 ▶). These results suggest that the conformation of the β2 strand affects CutA1 thermostability because hyperthermophilic CutA1s have no kink but mesophilic CutA1s have a kink, and psychrotrophic CutA1s have two short β strands divided. It is probable that hydrogen bonds between strands affect the stability.

Another peculiarity is that the β3 strand of SIB1–CutA1 is kinked by Pro residue (Pro58). Because Gln39 (between β2a and β2b) and Pro58 (β3) interact (Fig. 2*g* ▶), the kink in the β3 strand also seems to be one of the factors leading to the split of the β2 strand in SIB1–CutA1.

**Figure 3:**
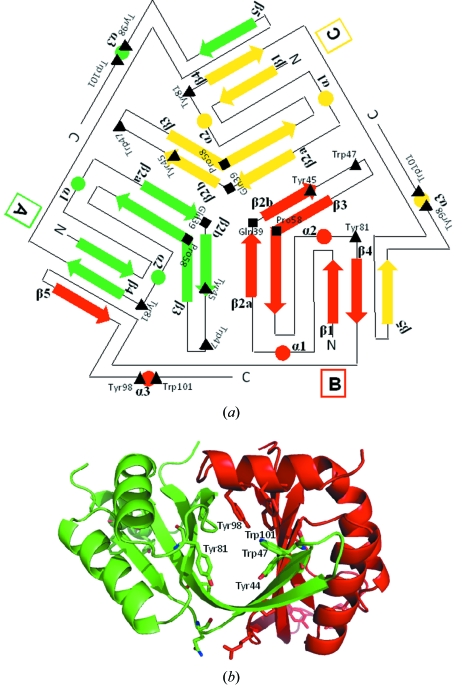
(*a*) Topological diagram of the trimeric structure of SIB1–CutA1. In the trimeric structure each monomer is coloured differently. Arrows and circles indicate β strands and α helices. Squares and triangles indicate Gln39/Pro58 and Tyr45/Trp47/Tyr81/Tyr98/Trp101. (*b*) Close-up view of the subunit interface. Each monomer is coloured differently. The highly conserved aromatic residues are shown.

SIB1–CutA1 subunit *A* (*B* or *C*) interacts with hydrogen bonds between β2a and β2b of subunit *C* (*A* or *B*), between β2b and β2a of subunit *B* (*C* or *A*) and between β4 and β5 of subunit *B* (*C* or *A*), resulting in a tightly intertwined trimer (Fig. 3*a* ▶). The intertwined interactions among β strands of SIB1–CutA1 seem to stabilize the trimeric structure as in other CutA1 proteins. In contrast to the β strands, three α helices are located on the outside of the trimer and cover β strands. In the subunit interfaces, highly conserved aromatic residues, Tyr45, Trp47 and Tyr81 from subunit *A* (*B* or *C*) and Tyr98 and Trp101 from subunit *B* (*C* or *A*), exist (Fig. 3*b* ▶) (see §3.3 below). These residues form a hydrophobic core at the trimer interfaces. This may also contribute to the stabilization of the CutA1 structures.

### 3.2. Stability of SIB1–CutA1

**Figure 4:**
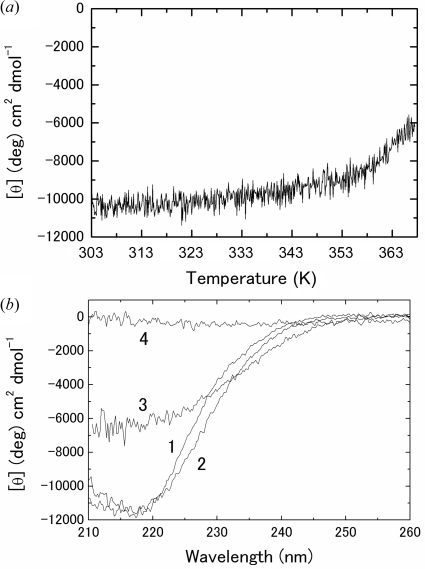
(*a*) Thermal denaturation curve of SIB1–CutA1 at pH 7.0. (*b*) CD spectra of SIB1–CutA1 at pH 7.0 and 293 K (1), pH 5.0 and 293 K (2), pH 7.0 and 368 K (3), and pH 5.0 and 368 K (4).

Fig. 4(*a*) ▶ plots the thermal denaturation of SIB1–CutA1 at pH 7.0. SIB1–CutA1 starts unfolding from around 353 K. However, the unfolding is not completed even at 368 K because the CD value at 368 K and pH 7.0 differs from that at 368 K and pH 5.0 (Fig. 4*b* ▶). *Shewanella* sp. SIB1 cannot grow at temperatures exceeding 303 K, and FKBP22 and ribo­nuclease HI from *Shewanella* sp. SIB1 are inactivated at less than 313 K (Suzuki *et al.*, 2005 ▶; Ohtani *et al.*, 2001 ▶). These results demonstrate the high stability of SIB1–CutA1. Differential scanning calorimetry (DSC) analysis enables us to provide thermal denaturation temperatures over 373 K. Unfortunately we could not obtain the thermal denaturation curve of SIB1–CutA1 by DSC because the solution contained DTT and EDTA, which are necessary for reversible unfolding of SIB1–CutA1.

Tanaka *et al.* (2006 ▶) have shown that the amino acid compositions of Ph-CutA1, Tt-CutA1 and Ec-CutA1 are quite different. There are fewer neutral residues (8.8%) but more charge residues (42.2%) in Ph-CutA1 as compared with those in Tt-CutA1 (21.4% and 28.2%) and Ec-CutA1 (25.9% and 22.3%). Furthermore, the number of intra-subunit ion pairs in Ph-CutA1, Tt-CutA1 and Ec-CutA1 is 30, 12 and 1. These properties are in (inverse) proportion to their stability: Ph-CutA1 unfolds at nearly 423 K, whereas the denaturation temperatures of Tt-CutA1 and Ec-CutA1 are 385.7 K and greater than 363 K (Sawano *et al.*, 2008 ▶). Here, the proportions of the neutral and charge residues in SIB1–CutA1 are 25.0% and 15.7%, and SIB1–CutA1 has two intra-subunit ion pairs. These results suggest that SIB1–CutA1 has a stability comparable with that of Ec-CutA1. It is noted that the denaturation temperature of Os-CutA1, which is from rice plant with an optimum growth temperature of 301 K, is close to 373 K at pH 7.0 (Sawano *et al.*, 2008 ▶).

### 3.3. High stability of the trimeric structural motif of CutA1

As described above, CutA1s have an unusually high stability that fundamentally depends on the optimal growth temperatures of their host organisms. The difference in stability is explained by the difference in electrostatic interactions and the conformation of the β2 strand. Here there is a question as to why the stability of CutA1 is so outstanding structurally and functionally.

From a structural viewpoint the high stability of CutA1 probably originates from the common trimer structural motif. The trimeric structure enables tightly intertwined interactions among the β strands. Tanaka *et al.* (2004 ▶) also pointed out hydrophobic interactions in Ph-CutA1 for stabilizing the trimeric structure. Furthermore, we found that the subunits in the trimer are firmly locked to each other with the highly conserved aromatic residues, such as Tyr45, Trp47, Tyr81, Tyr98 and Trp101, in SIB1–CutA1. These residues are located in the subunit interfaces. Therefore, we propose that CutA1 is stabilized by the aromatic cluster in addition to the β strands and hydrophobic interactions. We should note that some variant proteins in the highly conserved aromatic residues in So-CutA1 largely lose their stability (unpublished data). What is more, in order to clarify the protein stabilization mechanism we must consider such a structural motif in the future.

Since CutA1 unfolds at a remarkably higher temperature than the growth temperature of the host organisms and the stability depends on the growth temperature, CutA1 needs to maintain higher stability for this function. Usually, high thermostability of proteins correlates with high resistance against proteolysis. Bacterial CutA1 may have to continue capturing intracellular heavy metals without being degraded by intracellular proteases. In mammalian cells, CutA1 has been proposed to play a role in the transport of ligands to membranes (Perrier *et al.*, 2000 ▶) and in the folding, oligomerization and secretion of acetylcholinesterase (Liang *et al.*, 2009 ▶). In the present state we cannot explain the relation between the functions and stability of mammalian CutA1, but we believe that further investigations considering the high stability of CutA1 will be useful in obtaining critical insights into the functions of CutA1.

## Supplementary Material

PDB reference: 3ahp

## Figures and Tables

**Table 1:** Data collection and refinement statistics Values in parentheses are the highest-resolution bin of respective data.

Data collection	
Wavelength (Å)	1.0
Space group	*P*4_1_2_1_2
Unit cell (Å)	*a* = *b* = 134.67, *c* = 128.41
Resolution	50.0–2.7 (2.75–2.70)
No. of measured reflections	484499
No. of unique reflections	33103
Redundancy	14.6 (14.9)
*R*_merge_ (%)†	17.5 (77.2)
Completeness (%)	100 (100)
Average *I*/σ*I*	18.8 (3.03)
	
Refinement	
*R*_work_/*R*_free_ (%)‡	20.5/26.1
Total atoms included	5060
Water atoms included	61
R.m.s.d. bond length (Å)	0.01
R.m.s.d. bond angle (°)	1.312

† *R*_merge_ = Σ|*I*_*hkl*_ − 〈*I*_*hkl*_〉|/Σ*I*_*hkl*_, where *I*_*hkl*_ is the intensity measurement for reflection with indices *hkl* and 〈*I*_*hkl*_〉 is the mean intensity for multiply recorded reflections.

‡ *R*_work,free_ = Σ||*F*_obs_| − |*F*_calc_||/Σ|*F*_obs_|, where the *R*-factors are calculated using the working and free reflection sets, respectively. The free reflections comprise a random 10% of the data held aside for unbiased cross-validation throughout refinement.
